# Ratio of remnant cholesterol to high-density lipoprotein cholesterol in relation to gestational diabetes mellitus risk in early pregnancy among Korean women

**DOI:** 10.1371/journal.pone.0316934

**Published:** 2025-01-03

**Authors:** Jing Sheng, Chun-Fang Ma, Xiao-Fei Wu, Xiang-Xiang Li

**Affiliations:** 1 Department of Clinical Laboratory, Suzhou Ninth People’s Hospital Affiliated to Soochow University, Suzhou, Jiangsu, China; 2 Department of Emergency Medicine, Suzhou Ninth People’s Hospital Affiliated to Soochow University, Suzhou, Jiangsu, China; Zanjan University of Medical Sciences, ISLAMIC REPUBLIC OF IRAN

## Abstract

**Objective:**

There is no evidence to suggest that an association exists between the remnant cholesterol (RC) to high-density lipoprotein cholesterol (HDL-C) ratio and gestational diabetes mellitus (GDM). In this study, the RC/HDL-C ratio during the first trimester was examined as a potential indicator of the onset of GDM during the second trimester.

**Methods:**

This was a secondary analysis of data from a Korea-based prospective cohort study. The study involved 582 women within 14 weeks of pregnancy who were examined between November 2014 and July 2016 at two Korean hospitals. RC was calculated as total cholesterol (TC) minus the sum of low-density lipoprotein cholesterol (LDL-C) and HDL-C. The RC/HDL-C ratio was determined by dividing the RC content by the HDL-C content. The RC/HDL-C ratio and GDM occurrence were investigated utilizing a binary logistic regression model, various sensitivity analyses, and subgroup analyses. Additionally, the RC/HDL-C ratio was evaluated using receiver operating characteristic (ROC) analysis.

**Results:**

The average age of the pregnant women was 32.07 ± 3.78 years, and the RC/HDL-C ratio had a median value of 0.39. The prevalence of GDM was 6.01%. There was a positive association between the RC/HDL-C ratio and the incidence of GDM after adjusting for potential confounding variables (odds ratio: 21.78, 95% confidence interval [CI]: 3.55–133.73, *P* < 0.001). Furthermore, this association was validated by subgroup and sensitivity analyses. The results indicated that the RC/HDL-C ratio was a robust predictor of GDM, with an area under the ROC curve of 0.795 (95% CI: 0.723–0.868). The optimal threshold value was 0.45, with a sensitivity of 71.4% and a specificity of 75.3%. Compared with traditional lipid markers, including LDL-C, HDL-C, triglycerides, TC, and the emerging marker RC, the RC/HDL-C exhibited higher diagnostic efficacy.

**Conclusion:**

There is an increased risk of GDM associated with higher levels of the RC/HDL-C ratio between 12 and 14 weeks of gestation, independent of traditional risk factors. The RC/HDL-C ratio is more effective in diagnosing GDM than traditional lipid markers.

## Introduction

Gestational diabetes mellitus (GDM) is a glucose intolerance disease that is onset or first diagnosed during pregnancy. However, GDM does not meet the diagnostic criteria for diabetes in the general population [1]. Over the last couple of years, there has been a steady rise in the prevalence of GDM, ranging from 9.3% to 25.5%, potentially affecting 20 million newborns per year worldwide [2]. GDM poses various risks and complications for both the pregnant woman and the fetus. Generally, GDM increases the risk of preeclampsia, large-for-gestational-age newborns, shoulder dystocia, birth trauma, and neonatal hypoglycemia [3]. Mothers with GDM and their infants are more susceptible to developing obesity, cardiovascular disease (CVD), type 2 diabetes mellitus (T2DM), and other conditions [4]. Moreover, the diagnostic criteria for GDM vary worldwide and at different time nodes. Currently, the main international diagnostic criteria for GDM are the one- and two-step methods performed at 24–28 weeks of gestation [5]. The International Association of Diabetes and Pregnancy Study Groups (IADPSG) and the World Health Organization (WHO) support the one-step method, which has also received significant endorsement from multiple international organizations [6–8]. However, the American College of Obstetricians and Gynecologists (ACOG) prefers the two-step method, considering the potential increase in healthcare costs and the understanding that no particular screening strategy has been proven optimal [3]. However, a recent study found that GDM treatment during early pregnancy (< 20 weeks) effectively prevented the associated adverse outcomes [9, 10]. Consequently, early identification of pregnancies at risk of GDM is crucial for preventing adverse pregnancy outcomes and intergenerational transmission of metabolic dysregulation.

Abnormal serum lipid metabolism is a risk factor for GDM development. However, dyslipidemia during pregnancy can be a normal physiological phenomenon [11, 12]. It has been demonstrated that insulin resistance (IR) and estrogen stimulation during pregnancy can result in maternal hyperlipidemia [13]. Excessive eating and increased fat production result in the accumulation of maternal fat, generally occurring during the initial two trimesters. The process of fat storage is inhibited during the final trimester of pregnancy. This is due to increased activity of lipolytic enzymes and decreased lipoprotein lipase (LPL) activity in adipose tissue. This shift to a catabolic state favors the maternal use of lipids as an energy source and provides glucose and amino acids to the fetus. Therefore, it is unclear whether these changes in lipid metabolism are specific to pregnancy or can contribute to the emergence of future diseases. Due to the multifactorial etiology of GDM, its pathogenesis remains unclear [14]. However, the IR caused by obesity, inflammation, and oxidative stress is currently considered the primary pathogenic mechanism of GDM [15]. Hypertriglyceridemia and low levels of high-density lipoprotein cholesterol (HDL-C) are the two important metabolic abnormalities associated with IR [16].

Traditionally, HDL-C has been considered a lipid parameter against atherosclerosis (AS). Various studies indicate that HDL-C is closely associated with metabolic diseases [17, 18]. Multiple studies suggest that women with GDM have higher levels of circulating triglycerides (TG) and lower levels of HDL-C as compared to women without GDM [19, 20]. However, few studies have reported that the HDL-C concentration did not differ for women with and without GDM during pregnancy, and the relevant conclusions remain controversial [21, 22]. The clinical application of serum TG and its increase in GDM have been extensively investigated [23]. A new lipid marker—residual cholesterol (RC)—has recently been linked to CVD and other related disorders. Besides, the onset and progression of RC, diabetes mellitus (DM), CVD secondary to DM, and nonalcoholic fatty liver disease (NAFLD) have been investigated [24–26]. In the RC, lipoprotein cholesterol levels are normally determined by intermediate-density lipoprotein (IDL) and very low-density lipoprotein (VLDL) remnants in the fasting state (hepatocyte origin) and chylomicron (CM) remnants in the non-fasting state (intestinal origin) [27]. As the cholesterol content of partially lipolyzed triglyceride-rich lipoproteins (TRLs), RC may be more representative of circulating TG. Moreover, RC has been indicated to be more associated with IR than TG [27]. Meanwhile, RC, possibly as the most cholesterol-containing lipoprotein, may have a greater toxic effect on pancreatic beta cells (PBC) and promote their apoptosis [28]. Recently, the relationship between RC and GDM has been explored. However, related studies are limited [22, 29].

The RC/HDL-C ratio, as an emerging indicator of IR, has not been extensively studied, with previous investigations primarily focusing on its association with metabolic diseases. However, a relationship between the RC/HDL-C ratio and the risk of GDM has never been investigated. Based on the above related studies, we hypothesized that the RC/HDL-C ratio in early pregnancy may be correlated with the diagnosis of GDM. Given the similar IR pathway between GDM and T2DM, we conducted a secondary analysis [30] based on published research to evaluate the relationship between early pregnancy RC/HDL-C ratio and the possibility of developing GDM in Korean women in this study.

## Methods

### Data source

The primary data utilized in this study were freely accessed from the article titled "Nonalcoholic Fatty Liver Disease is a Risk Factor for Large-for-Gestational-Age Birthweight" by Lee SM et al., published in PLoS ONE (available at https://journals.plos.org/plosone) [30]. The publicly available primary data has been disseminated under the Creative Commons Attribution License, permitting unlimited use, distribution, and replication in any medium, provided that due acknowledgment is accorded to the author and the original source. We extend our sincere appreciation to the contributors of this invaluable data. The analysis included all the relevant data from November 2014 to July 2016.

### Study population

Seoul Metropolitan Government Seoul National University Boramae Medical Center and Incheon Seoul Women’s Hospital recruited 663 singleton pregnant women with less than 14 weeks of pregnancy. Data collection for the ongoing ’Fatty Liver in Pregnancy’ registry was conducted from November 2014 to July 2016 (ClinicalTrials. gov registration no. NCT02276144). Before participating, all individuals provided written consent as required by the original study. To ensure privacy protection, the researchers employed untraceable codes instead of identifiable participant information. The Institutional Review Board of the Seoul Metropolitan Government, Seoul National University Boramae Medical Center, and the Public Institutional Review Board of the Ministry of Health and Welfare of Korea approved the study [30]. This secondary analysis was based on a previously approved ethical framework; therefore, no additional ethical clearance was required. Moreover, the primary research was conducted following the Helsinki Declaration.

**Fig 1:**
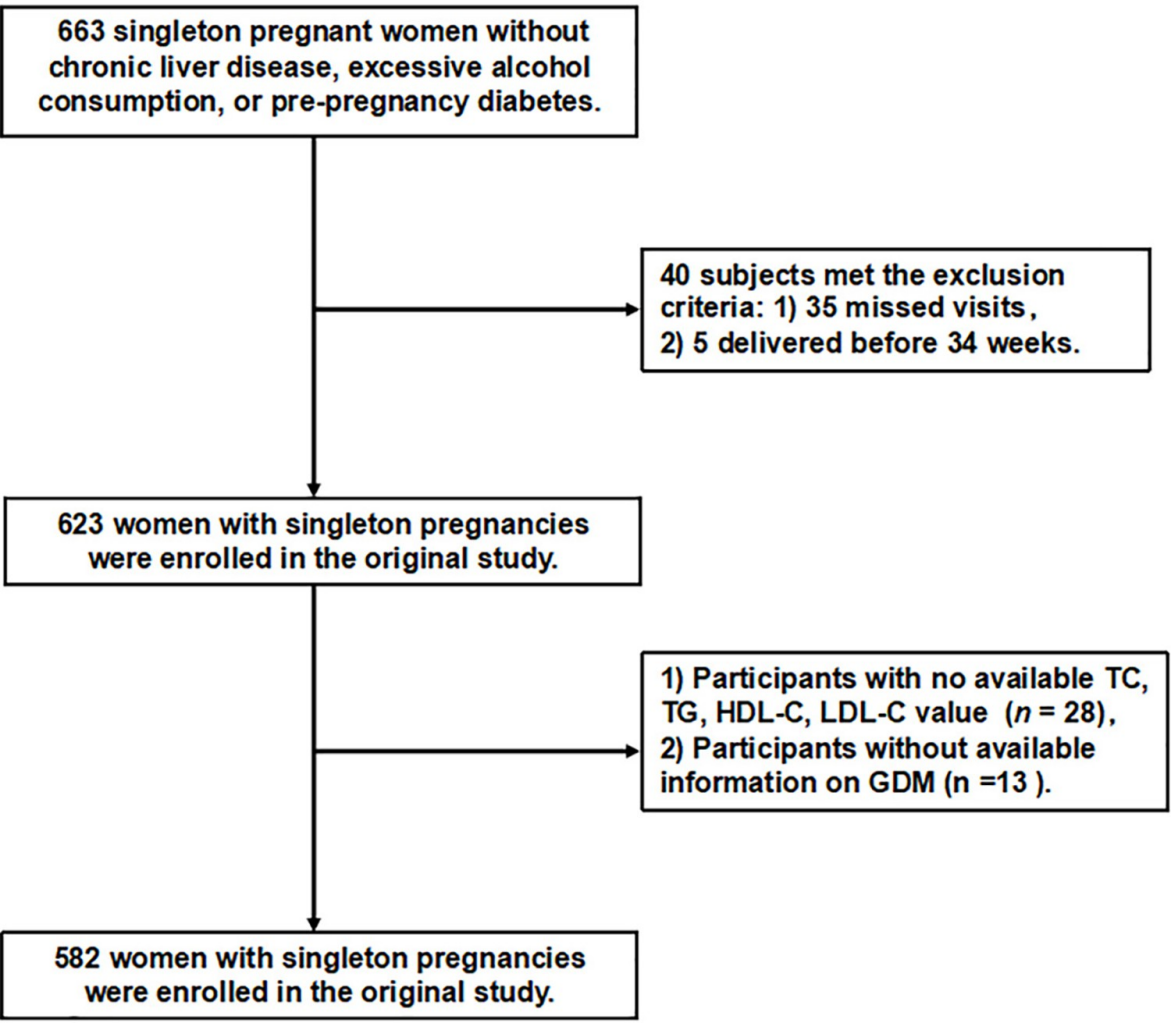
Flow chart of patient selection.

In the final analysis, patients with chronic liver disease, excessive alcohol consumption, and pre-gestational diabetes were excluded. Additionally, individuals who were lost to follow-up or experienced preterm birth before 34 weeks of gestation were excluded. Consequently, the preliminary investigation encompassed a cohort of 623 participants. In the present study, we eliminated missing data for total cholesterol (TC), TG, HDL-C, low-density lipoprotein cholesterol (LDL-C), insulin, pre-pregnancy body mass index (BMI), homeostasis model assessment of insulin resistance (HOMA-IR), aspartate aminotransferase (AST), and fasting plasma glucose (FPG) in 28 cases and incomplete information regarding GDM in 13 cases. Ultimately, the current investigation encompassed a total of 582 eligible participants, as depicted in **Fig 1**.

### Variables

The variables for the investigation were selected based on a comprehensive review of the initial investigation, clinical experience, and previous investigations into the risk factors associated with GDM. Due to this, we included the following covariates in our analysis: (1) categorical variables, such as parity and hepatic steatosis; (2) continuous variables, such as age, pre-pregnancy BMI, FPG, insulin levels, HOMA-IR (determined using the formula [insulin (IU/mL) × FPG (mmol/L)/22.5]), AST, alanine aminotransferase (ALT), gamma-glutamyl transferase (GGT), adiponectin levels, LDL-C, HDL-C, TG, and TC. The severity of hepatic steatosis was determined using a previously validated semiquantitative grading system, ranging from grades 0 to 3 [31]. Between the 10th and 14th weeks of gestation, a venous blood sample was obtained following a minimum fasting period of 8 h. The samples were centrifuged, aliquoted, and stored at –70°C for future analysis to evaluate hematological markers. FBG, routine lipid profiles, and liver enzyme concentrations were quantified using an enzymatic method (Glucose HK; Roche Diagnostics, Indianapolis, IN, USA) with a Roche/Hitachi 911 chemistry analyzer (Roche Diagnostics). Serum insulin concentrations were determined through an immunometric assay (IRMA) in duplicate using the same batch of a kit (INS-IRMA; DIAsource ImmunoAssays, Louvain-la-Neuve, Belgium). Adiponectin concentrations were quantified using an enzyme-linked immunosorbent assay (R&D Systems, United States).

### Ratio of RC to HDL-C

During the gestational period of 10 to 14 weeks, venous blood samples were analyzed for HDL-C and RC using a computerized analyzer after a minimum fasting period of 8 h. To obtain the RC/HDL-C ratio, the RC (mg/dL) was divided by HDL-C (mg/dL). RC (mg/dL) was calculated as follows: TC (mg/dL) ˗ HDL-C (mg/dL) ˗ LDL-C (mg/dL), as previously reported.

### Diagnosis of GDM

All participants underwent a two-step screening for GDM between 24 and 28 weeks of gestation, as recommended by the ACOG [32]. In non-fasting scenarios, pregnant women undergo a glucose challenge test (GCT) by ingesting a 50-gram oral glucose solution, after which serum glucose levels are assessed. A serum glucose level of 7.8 mmol/L or higher indicates a positive GCT. Individuals with a positive GCT underwent testing in the form of a 100-gram OGTT. For the diagnosis of GDM, at least two increased blood glucose levels must be confirmed: Fasting glucose level ≥ 5.3 mmol/L, 1 h postprandial level ≥ 10 mmol/L, 2 h postprandial level ≥ 8.6 mmol/L, and 3 h postprandial level ≥ 7.8 mmol/L.

### Data analysis

We partitioned the baseline data into three equal segments based on the RC/HDL-C ratio to evaluate its distribution. Continuous variables are represented as mean ± standard deviation or median (interquartile range), while categorical variables are presented as frequencies and percentages. Overall group differences were evaluated using either one-way analysis of variance for normally distributed data or the Kruskal–Wallis H test for non-normally distributed data.

Models of univariate and multivariate logistic regression were constructed in our study. For the non-adjusted model, no covariates were adjusted. The model I was adjusted for variables such as age, pre-pregnancy BMI, and parity. Adjusted model II incorporated variables such as age, pre-pregnancy BMI, parity, hepatic steatosis, levels of AST, GGT, ALT, adiponectin, and HOMA-IR. With corresponding 95% confidence intervals (CIs), the odds ratios (ORs) were adjusted to evaluate the GDM risk. Covariate adjustments were made based on a criterion where the inclusion of a covariate resulted in an OR change of at least 10%, which indicated its necessity for adjustment in the model.

This investigation utilized a sensitivity analysis to assess the robustness of the findings. To evaluate the correlation between the RC/HDL-C ratio and diverse factors and investigate possible non-linear relationships, we divided the RC/HDL-C ratio into three equal portions and computed the trend *p*-values. It is crucial to understand the interrelationships between obesity, NAFLD, and T2DM. To evaluate the correlation between the RC/HDL-C ratio and GDM, we excluded participants with hepatic steatosis grading greater than 0 or a pre-pregnancy BMI greater than 25 kg/m^2^.

To analyze subgroups, stratified logistic regression models were used in different subgroups, such as nulliparity, hepatic steatosis, age, pre-pregnancy BMI, and HOMA-IR. Clinical findings were used as cutoff points for categorical variables, while continuous variables were transformed into categorical variables based on median values or recognized clinical findings (age: below 35 or 35 years or above; pre-pregnancy BMI: below 25 or 25 kg/m^2^ or above; HOMA-IR: up to 2 or greater than 2). A stratification analysis was performed, with each stratum adjusted for all aforementioned factors, excluding the stratification factor itself. Furthermore, a log-likelihood ratio was used to determine heterogeneity in subgroup associations.

Moreover, a receiver operating characteristic (ROC) analysis was performed to assess the predictive capacity of the RC/HDL-C ratio, RC, TC, TG, LDL-C, HDL-C, and HOMA-IR for GDM. Subsequently, the sensitivity and specificity of a variable were evaluated through ROC analysis to determine its optimal threshold. R software (version 4.1.2) was used for statistical analysis. A two-tailed test was used to evaluate statistical significance, where results were considered statistically significant if *p*-values were below 0.05.

## Results

### Baseline characteristics

**Fig 2:**
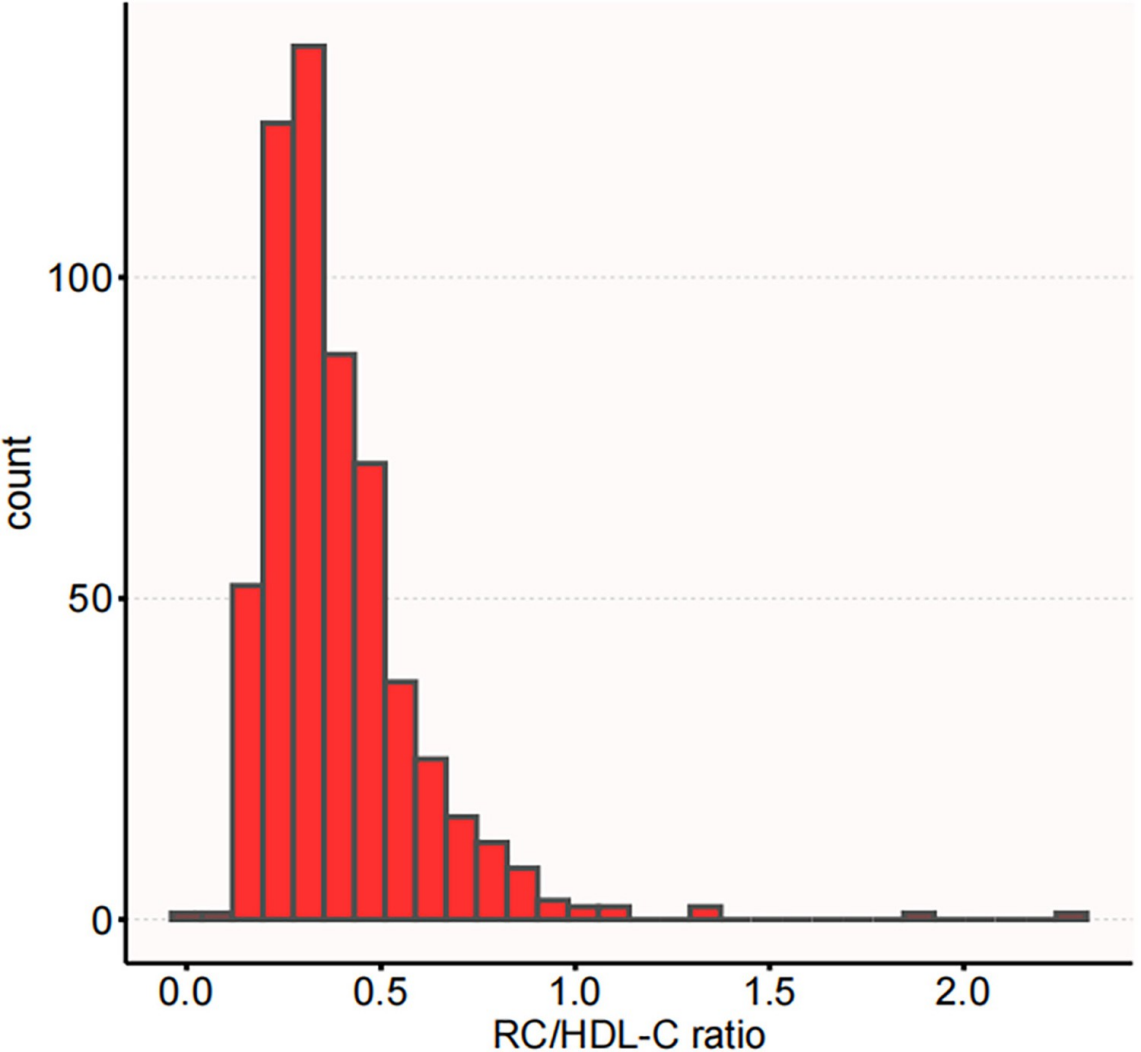
The distribution of RC/HDL-C ratio exhibited a skewed pattern. The distribution of RC/HDL-C ratio spans from 0.04 to 2.31, with a median (interquartile range) of 0.33 (0.26, 0.47).

The demographic and clinical characteristics of the study participants are provided in **Table 1**. The average age observed was 32.07 ± 3.78 years. Among the participants, 278 individuals (47.77%) had multiparous status, while 472 individuals (81.10%) were free from fatty liver disease. GDM was diagnosed in 35 (6.01%) pregnant women. Participants were classified into three categories based on their levels of the RC/HDL-C ratio. The highest tertile group exhibited significantly higher levels of pre-pregnancy BMI, TG, GGT, LDL-C, RC, and HOMA-IR levels than the lowest. Conversely, HDL-C and adipokines demonstrated an inverse trend. The distribution of RC/HDL-C ratio displayed a positively skewed pattern, with a median value of 0.33 (interquartile range: 0.26–0.47), as depicted in **Fig 2**.

**Table 1:** The baseline characteristics of participants.

Variables	Total (582)	T1(≤0.28, 194)	T2 (0.28 -≤0.42, 194)	T3 (>0.42, 194)	*P* value
**Age, years**	32.07 ± 3.78	31.56 ± 3.57	32.48 ± 3.56	32.17 ± 4.14	0.050
Pre-pregnancy BMI (kg/m^2^)	22.03 ± 3.49	21.24 ± 2.98	22.09 ± 3.53	22.78 ± 3.76	< 0.001
**Parity, n (%)**					0.082
**No**	304 (52.23)	114 (58.76)	94 (48.45)	96 (49.48)	
**Yes**	278 (47.77)	80 (41.24)	100 (51.55)	98 (50.52)	
**Hepatic steatosis, n (%)**					< 0.001
**Grade 0**	472 (81.10)	170 (87.63)	167 (86.08)	135 (69.59)	
**Grade 1**	85 (14.60)	23 (11.86)	21 (10.82)	41 (21.13)	
**Grade 2**	17 (2.92)	1 (0.52)	4 (2.06)	12 (6.19)	
**Grade 3**	8 (1.37)	0 (0)	2 (1.03)	6 (3.09)	
**AST (IU/L)**	16.00 (14.00, 20.00)	16.00 (14.00, 18.75)	16.00 (14.00, 19.00)	17.00 (14.00, 21.00)	0.162
**ALT (IU/L)**	11.00 (8.00, 15.00)	11.00 (8.00, 13.75)	11.00 (8.00, 15.00)	12.00 (8.00, 18.00)	0.098
**GGT(IU/L)**	12.00 (10.00, 15.00)	11.00 (10.00, 14.00)	12.00 (10.00, 15.00)	13.00 (10.00, 17.00)	0.002
**Adipokines(ng/mL)**	5026.25 (2821.33, 8051.02)	6588.00 (3778.50, 10005.25)	5331.05 (3310.75, 8236.70)	3278.60 (1941.53, 6069.62)	< 0.001
**TC (mg/dL)**	172.85 ± 27.04	171.51 ± 26.67	171.78 ± 26.27	175.27 ± 28.12	0.312
**TG (mg/dL)**	119.00 ± 47.56	81.96 ± 19.14	110.70 ± 21.63	164.34 ± 49.60	< 0.001
**HDL-C(mg/dL)**	65.02 ± 13.35	74.08 ± 11.82	64.92 ± 10.91	56.05 ± 10.68	< 0.001
**LDL-C(mg/dL)**	84.04 ± 21.62	81.05 ± 21.04	84.72 ± 20.08	86.35 ± 23.38	0.047
**FPG (mg/dL)**	76.88 ± 9.66	76.78 ± 10.11	76.73 ± 8.62	77.12 ± 10.22	0.909
**Insulin (μIU/mL)**	8.40 (5.32, 11.57)	6.40 (4.30, 9.47)	7.90 (5.50, 10.88)	10.65 (7.43, 15.30)	< 0.001
**HOMA-IR**	1.50 (1.00, 2.30)	1.20 (0.80, 1.87)	1.50 (1.00, 2.20)	2.00 (1.40, 2.88)	< 0.001
**RC (mg/dL)**	23.80 ± 9.52	16.38 ± 3.81	22.14 ± 4.33	32.87 ± 9.92	< 0.001
**RC/HDL-C ratio**	0.33 (0.26, 0.47)	0.23 (0.19, 0.26)	0.33 (0.31, 0.37)	0.54 (0.47, 0.66)	< 0.001

Continuous variables are presented as mean (standard deviation) or medians (interquartile range), while categorical variables are expressed as percentages (%).

RC remnant cholesterol, BMI body mass index, ALT alanine aminotransferase, AST aspartate aminotransferase, GGT gamma-glutamyl transferase, HDL-C high-density lipoprotein cholesterol, TC total cholesterol, TG triglycerides, LDL-C low-density lipid cholesterol, FPG fasting plasma glucose, HOMA-IR homeostasis model assessment-insulin resistance.

### The outcomes of univariate analyses

The results of the univariate logistic analysis are presented in **Table 2**. These results indicated a positive correlation between pre-pregnancy BMI, liver steatosis grade, TC levels, TG levels, ALT levels, GGT levels, FPG levels, insulin levels, HOMA-IR index, and RC/HDL-C ratio, and the occurrence of GDM. Additionally, GDM incidence exhibited a negative correlation with HDL-C levels.

**Table 2:** The outcomes of the univariate analysis.

Variables	OR (95% CI)	*P*-value
**Age, years**	1.038 (0.948~1.137)	0.420
**Pre-pregnancy BMI (kg/m2)**	1.279 (1.177~1.389)	<0.001
**Parity, n (%)**		
**No**	ref	
**Yes**	1.035 (0.522~2.05)	0.922
**Hepatic steatosis, n (%)**		
**Grade 0**	ref	
**Grade 1**	3.608 (1.525~8.537)	0.004
**Grade 2**	27.081 (9.175~79.937)	<0.001
**Grade 3**	18.28 (3.994~83.662)	<0.001
**AST (IU/L)**	1.019 (0.992~1.046)	0.169
**ALT (IU/L)**	1.039 (1.015~1.063)	0.001
**GGT(IU/L)**	1.035 (1.008~1.062)	0.010
**Adipokines(ng/mL)**	0.999 (0.999~1)	<0.001
**TC (mg/dL)**	1.019 (1.013~1.026)	<0.001
**TG (mg/dL)**	5.244 (2.606~10.552)	<0.001
**HDL-**C**(mg/dL)**	0.97 (0.944~0.997)	0.032
**LDL-**C**(mg/dL)**	0.998 (0.982~1.014)	0.787
**FPG (mg/dL)**	1.069 (1.036~1.103)	<0.001
**Insulin (μIU/mL)**	1.118 (1.068~1.171)	<0.001
**HOMA-IR**	1.482 (1.225~1.792)	<0.001
**RC (mg/dL)**	1.1 (1.065~1.136)	<0.001
**RC/HDL-C ratio**	57.472 (13.318~248.008)	<0.001

OR, odds ratio; CI, confidence interval.

RC remnant cholesterol, BMI body mass index, ALT alanine aminotransferase, AST aspartate aminotransferase, GGT gamma-glutamyl transferase, HDL-C high-density lipoprotein cholesterol, TC total cholesterol, TG triglycerides, LDL-C low-density lipid cholesterol, FPG fasting plasma glucose, HOMA-IR homeostasis model assessment-insulin resistance.

### The outcomes of multivariate analyses

A multivariate logistic regression model was used to determine the association between the RC/HDL-C ratio and the incidence of GDM (**Table 3**). The OR of the unadjusted model was 57.47, with a 95% CI of 13.32 to 248.01 (*P* < 0.001). In Model I, the results consistently exhibited stable outcomes without significant variations (OR: 41.24, 95% CI: 8.63–197.16, *P* < 0.001). Furthermore, even in Model II, there was a statistically significant association between the RC/HDL-C ratio and the incidence of GDM (OR: 21.78, 95% CI: 3.55–133.73, *P* < 0.001). The adjustment factors incorporated in each of Model I and Model II are expressed in the data analysis section above and can be seen in the table notes.

**Table 3:** Association of RC/HDL-C ratio with the incidence of GDM across various models.

	Non-adjusted model		Model I		Model II	
	OR [95% CI]	*P* value	OR [95% CI]	*P* value	OR [95% CI]	*P* value
**RC/HDL-C ratio**	57.47 (13.32~248.01)	<0.001	41.24 (8.63~197.16)	<0.001	21.78 (3.55~133.73)	0.001
**RC/HDL-C ratio tertile**						
**T1**	Reference	-	Reference	-	Reference	-
**T2**	2.38 (0.61~9.35)	0.213	1.84 (0.45~7.59)	0.396	1.67 (0.37~7.47)	0.501
**T3**	9.42 (2.79~31.74)	<0.001	6.91 (1.98~24.15)	0.002	4.11 (1.05~16.12)	0.043
*P* for trend		<0.001		<0.001		0.017

Data are presented as OR [95% CI].

Model I adjusted for adjusted age, pre-pregnancy BMI, and parity.

Model II adjusted for age, pre-pregnancy BMI, parity, hepatic steatosis, AST, GGT, ALT, adiponectin, and HOMA-IR.

### Sensitive analysis

As presented in **Table 4,** supplementary sensitivity analyses were performed specifically on participants with a BMI less than 25 kg/m^2^. When controlling for confounding variables, a significant positive correlation was found between RC/HDL-C ratio and the incidence of GDM (OR = 22.13, 95% CI: 1.51–324.71). Furthermore, we conducted sensitivity analyses by including individuals without any signs of hepatic steatosis (grade 0). After adjusting for covariates, including age, parity, pre-pregnancy BMI, AST, GGT, ALT, HOMA-IR, and adiponectin, the findings consistently indicated an association between the RC/HDL-C ratio and GDM incidence (OR = 32.28, 95% CI: 2.08–500.67). Sensitivity analysis revealed robust and reliable results.

**Table 4:** Association of RC/HDL-C ratio with the incidence of GDM in different sensitivity analyses.

Exposure	Model I		Model II	
	OR [95% CI]	*P* value	OR [95% CI]	*P* value
**RC/HDL-C ratio**	22.13 (1.51~324.71)	0.024	32.28 (2.08~500.67)	0.013
**RC/HDL-C ratio tertile**				
**T1**	Reference	-	Reference	-
**T2**	0.35 (0.03~4.12)	0.404	0.27 (0.02~3.55)	0.322
**T3**	3.67 (0.69~19.59)	0.129	3.29 (0.65~16.61)	0.150
*P* for trend		0.053		0.037

Data are presented as OR [95% CI].

Model I presents a sensitivity analysis that excludes individuals with pre-pregnancy BMI of ≥ 25 kg/m^2^, adjusting for age, parity, hepatic steatosis, AST, GGT, ALT, total cholesterol, LDL-C, HOMA-IR, and adiponectin.

Model II presents a sensitivity analysis that excludes individuals with grade 1–3 hepatic steatosis, adjusting for age, parity, hepatic steatosis, AST, GGT, ALT, total cholesterol, LDL-C, HOMA-IR, and adiponectin.

### Subgroup analysis

**Fig 3:**
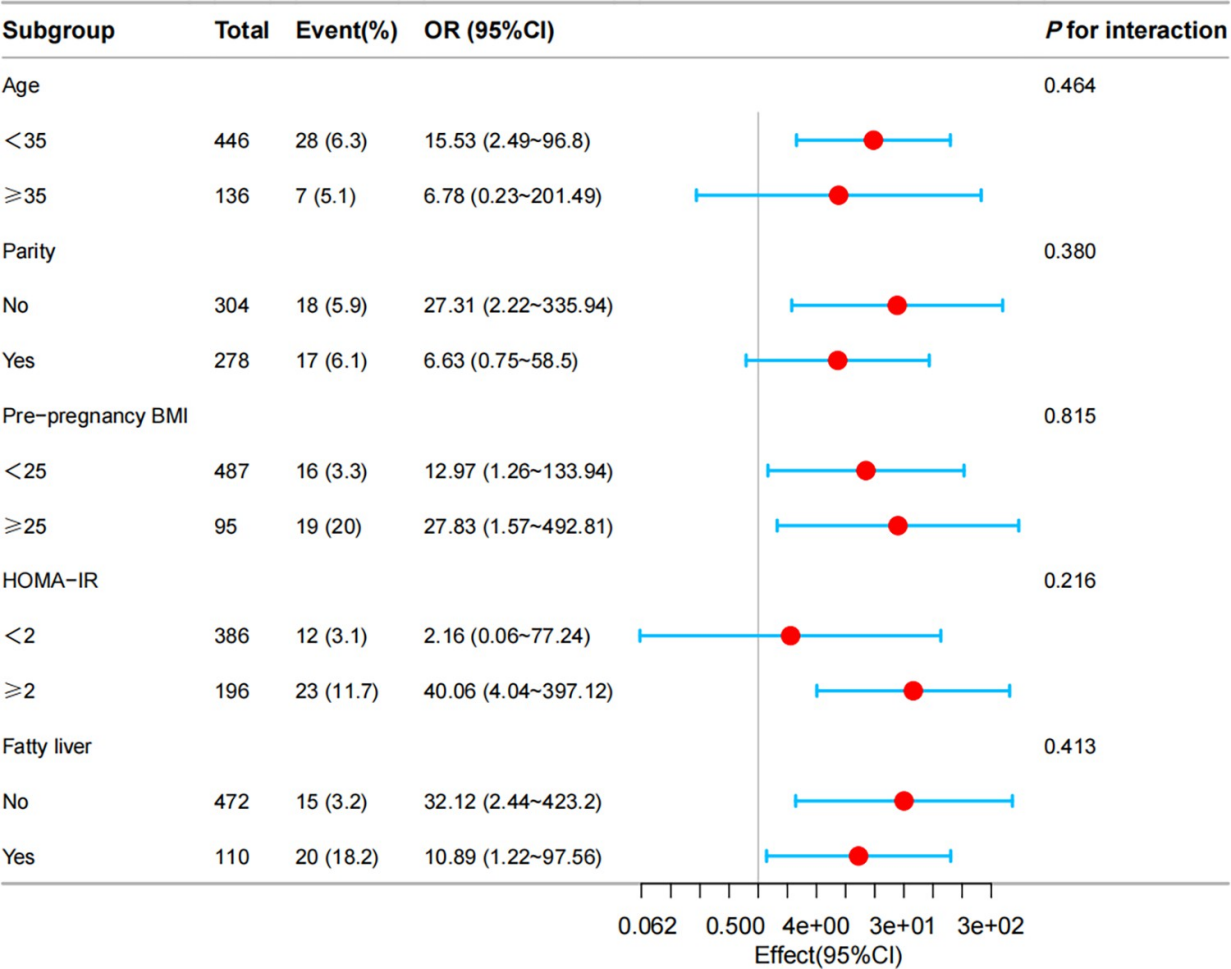
Forest plots for RC/HDL-C ratio subgroup analyses with the incidence of GDM. Multivariable logistic regression analyses were performed across various populations, with adjustments made for age, pre-pregnancy BMI, parity, hepatic steatosis, AST, GGT, ALT, adiponectin, and HOMA-IR.

To investigate the potential factors that could affect the association between the RC/HDL-C ratio and the incidence of GDM, we performed a subgroup analysis, as demonstrated in **Fig 3**. Pre-pregnancy BMI, hepatic steatosis, age, parity, and HOMA-IR were stratified. Even after accounting for the potential confounders listed previously, the robust association between the RC/HDL-C ratio and GDM risk remained evident. Furthermore, the robustness of our results was validated through subgroup analysis.

### ROC analysis

**Fig 4:**
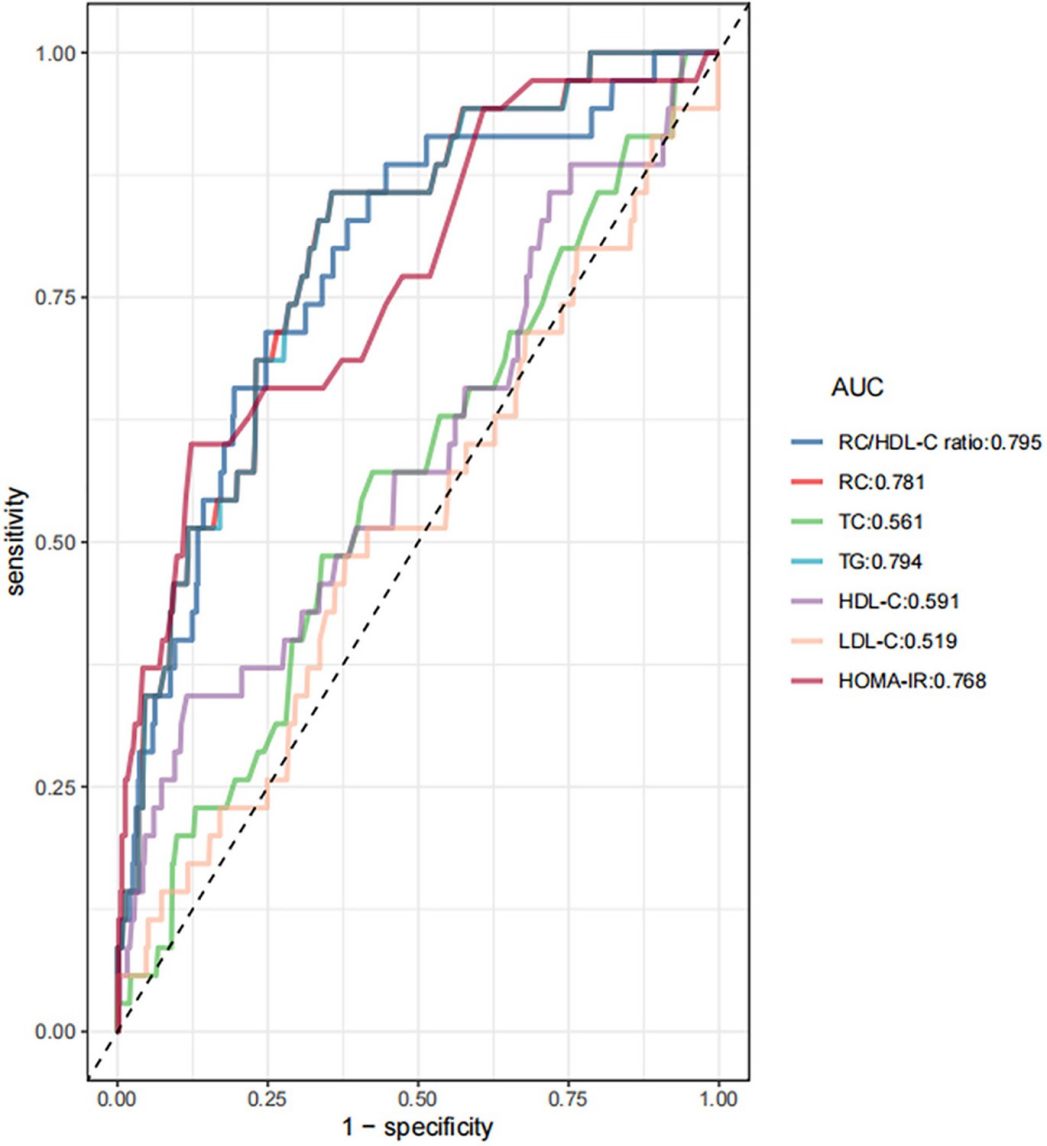
The RC/HDL-C ratio was assessed for its predictive capacity of GDM across all participants using ROC analysis. The analysis revealed that the AUC for the RC/HDL-C ratio was 0.795. This metric outperformed traditional markers such as RC, TG, HDL-C, TC, LDL-C, and HOMA-IR in predicting GDM.

ROC curve analysis was used to evaluate the prognostic efficacy of the RC/HDL-C ratio in the prediction of GDM. The findings indicated that the area under the curve (AUC) for the RC/HDL-C ratio was 0.795 (95% CI: 0.723–0.868), as detailed in **Table 5** and displayed in **Fig 4**. Generally, the RC/HDL-C ratio exhibits superior predictive accuracy for GDM compared to other biomarkers, including TG, TC, HDL-C, LDL-C, RC, and HOMA-IR, with an anticipated higher AUC. An analysis utilizing Youden’s index identified a threshold of 0.45 for the RC/HDL-C ratio, yielding optimal specificity and sensitivity values of 71.4% and 75.3%, respectively, GDM prediction.

**Table 5:** Receiver operating characteristic curve areas for each lipid parameter in the identification of GDM.

	AUC	95%CI low	95%CI up	Best threshold	Specificity	Sensitivity
**RC/HDL-C ratio**	0.795	0.723	0.868	0.45	0.753	0.714
**RC(mg/dL)**	0.781	0.700	0.862	24.3	0.644	0.857
**TC(mg/dL)**	0.561	0.461	0.661	174.5	0.576	0.571
**TG(mg/dL)**	0.794	0.722	0.867	121.5	0.644	0.857
**HDL-C(mg/dL)**	0.591	0.484	0.698	49.2	0.885	0.343
**LDL-C(mg/dL)**	0.519	0.413	0.625	77.55	0.622	0.486
**HOMA-IR**	0.768	0.680	0.856	2.75	0.877	0.600

AUC area under the curve, CI confidence interval; RC remnant cholesterol, HDL-C high-density lipoprotein cholesterol, TC total cholesterol, TG triglycerides, LDL-C low-density lipid cholesterol, HOMA-IR homeostasis model assessment-insulin resistance.

## Discussion

According to a secondary analysis of Korean prospective data, the RC/HDL-C ratio was independently associated with the risk of GDM in the second trimester. Moreover, a sensitivity analysis confirmed the stable association between the RC/HDL-C ratio and GDM risk. Furthermore, the RC/HDL-C ratio can predict GDM with an AUC of 0.795 (95% CI: 0.723–0.868), a sensitivity of 71.4%, and a specificity of 75.3% when the cutoff value is 0.45. The RC/HDL-C ratio has greater predictive power than conventional lipid indices, including TG, LDL-C, HDL-C, and the emerging RC lipid index for GDM in early pregnancy. Therefore, this new marker may be a straightforward and non-invasive diagnostic index of GDM and useful for early diagnosis, treatment, and prognosis.

GDM is fundamentally a heterogeneous disease [33] that develops during pregnancy. According to previous studies, there is a linear relationship between increased blood glucose levels during 24–28 weeks of gestation and adverse perinatal outcomes [34]. However, an unclear inflection point suggests we should focus on blood glucose levels in early pregnancy. According to the WHO diagnostic criteria for GDM [7], women diagnosed with diabetes in early pregnancy are more likely to have adverse outcomes and require insulin or other glucose-lowering medications than those generally diagnosed at 24–28 weeks of gestation [35]. A meta-analysis revealed that, with and only in the first trimester of pregnancy, intervention can be effective in treating GDM [36]. A large randomized controlled trial in an Australian population, published in the latest New England Journal of Medicine [9], reported that treating GDM immediately before 20 weeks’ gestation resulted in a slightly lower combined incidence of poor neonatal prognosis than that if no treatment was administered, making early diagnosis urgent. However, according to the diagnosis of GDM based on blood glucose in early pregnancy, the highest rate of positive GDM in the second trimester is only about 50%, which suggests a possibility of excessive diagnosis and treatment [7, 37]. Consequently, it is imperative to find an efficient and stable economic surveillance indicator to diagnose GDM despite uncertainty about the benefits and harms of early intervention.

Traditional lipid indicators are extensively used in clinical practice. Studies have shown that non-traditional lipid parameters, including the atherosclerotic index (AIP), RC, and non-high-density lipoprotein cholesterol (non-HDL-C), have higher predictive value in identifying abnormal glucose metabolism in patients with GDM [22, 38, 39]. The advantage of these non-traditional parameters is their ability to provide a more comprehensive assessment of metabolic status, especially during pregnancy, when physiological changes in women can affect the accuracy of traditional indicators. The progress of lipidomics provides a new perspective on understanding the pathophysiological mechanisms of GDM. In prospective studies, multiple fatty acids, phospholipids, lipoproteins, certain glycerolipids, and cholesterol have been reported to be associated with incident GDM [15]. The elevation of certain lipid species, including TG and cholesteryl esters, is closely associated with GDM [40]. Moreover, lipidomics studies have indicated that the lipid profiles of patients with GDM are associated with the risk of developing T2DM in the future, suggesting that abnormalities in lipid metabolism can play an important role in GDM pathophysiology [41]. However, different lipidomics platforms result in difficulties in external validation and expensive assays, which pose limitations for the field of lipidomics.

Reducing serum LDL-C levels is the primary therapeutic target for the primary and secondary prevention of CVD. Nevertheless, patients with substantial decreases in LDL-C levels have a considerable risk of CVD, namely residual risk. Accordingly, high levels of RC can partially explain residual risk [42] and are independent of the conventional lipid profile. Numerous epidemiological studies [43] indicated that the RC concentration is positively associated with the occurrence of cardiovascular events such as AS. Furthermore, RC has stronger all-cause mortality in ischemic heart disease than the conventional lipid profile [44]. Related mechanisms can include the following: (1) Through the LPL-mediated (and, to a lesser extent, hepatic lipase-mediated) removal of TG and cholesteryl ester transfer protein-mediated cholesterol exchange in the LDL and HDL, RC particles contain more cholesterol [45] than the nascent CM or VLDL, and RC is larger than LDL-C particles and carries more cholesterol (possibly 5–20 times more than LDL) [46]. Cholesterol is considered the primary component of AS. (2) Unlike LDL, RC [47] is absorbed by macrophages and smooth muscle cells. (3) RC can rapidly enter the subendothelial space, with RC efflux being slow compared to the rate at which they enter, increasing the chance of macrophage internalization and foam cell formation [48]. (4) As with adipocytes and cardiomyocytes, atherosclerotic lesional macrophages can produce large amounts of LPL. The LPL-mediated release of free fatty acids from the accumulated RC can induce the production of pro-inflammatory mediators (cytokines, interleukins, and adhesion molecules); therefore, accelerating the recruitment of leukocytes to areas of inflammation. Simultaneously, we discovered that increased RC levels were linked to reduced inflammation and ischemic heart disease, suggesting that RC causes atherosclerotic with inflammatory components [49]. Several Mendelian randomization analyses have been published, providing a genetic basis for the causal role of TRLs (RC precursors) in AS [50]. Concurrently, more studies have focused on RC, NAFLD, and T2DM metabolic diseases. NAFLD is a hepatic manifestation of the metabolic syndrome. Conventional dyslipidemia, characterized by elevated serum TG and LDL-C levels and decreased HDL-C levels, has been identified as a risk factor for NAFLD [51]. Decreased LPL activity results in insufficient clearance of TRLs. The activation of LPL has been confirmed to reduce the severity of hepatic steatosis effectively [49]. Second, low-grade systemic inflammation, which plays a key role in NAFLD pathogenesis, can be involved in RC and NAFLD. In the second trimester, NAFLD is predictive of GDM, indicating that RC may be involved in the pathogenesis of NAFLD to GDM and that IR may be a significant mechanism [52]. Similarly, diabetic dyslipidemia contributes to the progression of diabetes at an early stage. With the finding that statins do not control diabetic dyslipidemia [53], the focus of research has shifted from traditional lipid profiles such as LDL-C to TRLs. Moreover, the fact that RC has reportedly been associated with the risk of residual CVD in diabetes sidesteps this point [25]. The RC is the cholesterol content of TRLs. Although plasma TG can be used as a surrogate marker for clinical RC, it represents different types of lipid disorders. RC can promote IR more directly than TG, while the toxic effect of cholesterol on PBC leads to the structural changes of insulin-containing granules [28]. A study with a large sample in China reported that RC transcends LDL-C and is associated with diabetes, which can be mediated by IR and proinflammatory status [24]. Moreover, diabetes is more likely to develop in women exposed to RC, which can be associated with different dietary profiles, estrogen, and cholesterol metabolism. At the genomic level, GDM and T2DM have a genetic correlation. Several studies have indicated that the genetics of GDM risk can be classified into two groups: one is T2DM risk, and the other is the main factor specific to GDM. The GDM-specific mechanism can be associated with islet cells, central glucose homeostasis, steroidogenesis, and placental expression [54]. It suggests that GDM and T2DM seek differences in gene mechanisms.

Research indicates maternal lipid profiles, particularly TG and RC, can significantly influence fetal growth and metabolic outcomes. For instance, a study found that higher maternal TG and RC levels were associated with increased fetal head circumference and abdominal circumference growth rates, suggesting a direct impact on fetal development. Furthermore, these lipid levels were positively correlated with the risk of large-for-gestational-age infants, suggesting that dysregulated lipid metabolism can affect maternal and fetal health [55]. The future of lipid components other than TG in GDM is evolving and controversial [56–58]. Currently, there is limited research on the relationship between RC and GDM. Moreover, the relevant literature is quite scarce, and the metabolic mechanisms need further investigation. Weiming Wang et al. [29] conducted a prospective study on the relationship between GDM and RC using the RC calculation method. Additionally, the experiment was nested and measured relevant parameters in Chinese women in the first trimester (12–14 weeks), which demonstrated that increased RC levels were associated with an increased risk of GDM despite traditional risk factors. Pregnant women with elevated BMI, TG, and RC had a significantly increased risk of GDM. Our study found that RC can be correlated with GDM risk. Additionally, further analysis indicated that RC/HDL-C ratio was an independent risk factor for GDM, which persisted even after adjusting for insulin, fasting glucose, and hepatic steatosis. However, the study by Weiming Wang et al. did not incorporate any of these metrics to explore. The mechanisms by which RC affects GDM may involve inflammatory pathways and endothelial dysfunction. Increased RC can lead to the activation of pro-inflammatory pathways, which can exacerbate IR and contribute to the pathophysiology of GDM. Additionally, maternal dyslipidemia, characterized by elevated cholesterol levels, has been associated with impaired vascular function in the placenta, potentially affecting nutrient delivery to the fetus and leading to adverse outcomes [59]. Moreover, studies utilizing Mendelian randomization have provided causal evidence supporting the role of RC in in GDM development. These analyses suggest that genetically predicted elevated RC levels are associated with an increased risk of GDM, highlighting the importance of lipid management during pregnancy [60]. In summary, the interplay between RC and GDM involves metabolic, inflammatory, and vascular mechanisms.

Ethnic differences in lipid metabolism and GDM risk are critical areas of research that highlight how genetic, environmental, and lifestyle factors interact to influence metabolic health across diverse populations. Research indicates that South Asian women tend to have a higher prevalence of GDM compared to their White European counterparts, which can be attributed to differences in body fat distribution and insulin sensitivity at a given BMI level [61]. This ethnic disparity is further complicated by the fact that South Asians often exhibit a more adverse lipid profile, including higher levels of TG and lower levels of HDL-C, which are risk factors for metabolic diseases [62, 63]. Moreover, genetic factors significantly influence lipid metabolism and GDM risk. Variants in genes associated with lipid metabolism, including the cholesteryl ester transfer protein and LPL, have been shown to influence lipid levels differently across ethnic groups. Studies have demonstrated that black South African women exhibit a more favorable lipid profile than their white counterparts, a phenomenon that is associated with particular genetic polymorphisms [62]. Besides, a study on the Korean population reported that the variants in KCNQ1 were associated with a risk for GDM and decreased insulin secretion capacity [64]. Insufficient insulin secretion can enhance lipolysis, resulting in elevated concentrations of TG and cholesterol in the bloodstream. This suggests that genetic predisposition can contribute to the observed ethnic differences in lipid metabolism and the associated risk of GDM. In conclusion, understanding the ethnic differences in lipid metabolism and their implications for GDM risk is essential for developing targeted prevention and treatment strategies.

HDL-C is involved in the reverse transport of cholesterol in the human body, where it can "remove" cholesterol from the atherosclerotic vessel wall and transport it to the liver for metabolic clearance. Generally, HDL-C reduction is a characteristic dyslipidemia of T2DM [65]. Researchers reported that a low HDL-C level affects glucose homeostasis by decreasing insulin secretion, insulin sensitivity, and adenosine monophosphate-activated protein kinase (AMPK) activity [66]. With further studies of HDL, several additional functional features were identified, including antioxidant [67] and anti-inflammatory properties [68]. The anti-inflammatory effect of HDL plays a role in preventing T2DM [68]. Reactive oxygen species can directly damage PBC, interfere with the normal function of the insulin signaling pathway, and reduce cell responsiveness to insulin, leading to increased blood glucose levels. The HDL-associated antioxidant enzyme paraoxonase 1 (PON1) enhances the scavenging of lipid hydroperoxides, thereby reducing oxidative stress. According to experimental evidence, this increases insulin secretion in mice and cellular models [69].

Generally, a normal increase in HDL-C content is observed from the first trimester through the second trimester. However, a decrease is observed in the third trimester. HDL-C concentrations were lower throughout GDM [13]. There is evidence that moderate increases in HDL-C concentration can protect against GDM, and HDL-C levels are inversely correlated with GDM risk [19]. According to Jin et al. [70], relatively low maternal HDL-C was associated with an increased risk of both GDM and macrosomia, while high HDL-C was protective. However, we observed that HDL-C was low in GDM. Moreover, univariate analysis exhibited a negative correlation with the occurrence of GDM. HDL-C is involved in modulating insulin sensitivity. Lower levels of HDL-C are associated with increased IR, a key factor in GDM development. This relationship can arise from HDL-C’s role in influencing peripheral glucose uptake, promoting beneficial signaling pathways that enhance insulin action [71]. First, oxidative stress and inflammation are elevated during pregnancy, and these factors are further exacerbated in GDM. HDL-C has anti-inflammatory and antioxidant properties that can reduce these conditions. Studies have indicated that HDL-C can influence the activity of PON1, an enzyme associated with antioxidant properties. In GDM, decreased PON1 activity correlates with lower HDL-C levels, indicating a disturbance in antioxidative mechanisms that may contribute to IR development [72]. Second, HDL-C is crucial for cholesterol efflux and lipid homeostasis. Impairments in these functions can result in the accumulation of lipotoxic intermediates, further exacerbating IR and promoting PBC dysfunction—both critical in GDM pathogenesis [73]. Furthermore, HDL-C plays a role in maintaining endothelial function and vascular health. Reduced HDL-C levels can compromise endothelial integrity, affecting placental blood flow and glucose transport and contributing to GDM pathophysiology [23]. The relationship between HDL-C and GDM is complex and involves lipid metabolism, inflammatory responses, and oxidative stress.

Given the preceding positive correlation of RC with the occurrence of related diseases and the negative correlation of HDL-C, this study investigates whether the combination of RC and HDL-C (RC/HDL-C ratio) can improve the predictive power for GDM. Currently, RC/HDL-C ratio-based studies in related diseases are limited. Reportedly, the RC/HDL-C ratio contributes to and mediates the risk of BMI-related NAFLD and contributes more to the mediation effect than conventional lipid markers [26]. The same results have been studied and verified in the Japanese population [74]. Meanwhile, the RC/HDL-C ratio can reflect the balance between potential pro-atherogenic lipoprotein particles and is a useful independent predictor of myocardial damage in some patients with DM [75].

Our study is the first prospective study on the relationship between RC/HDL-C ratio and GDM. We found a positive correlation between the RC/HDL-C ratio and GDM risk. The risk remained significant after adjustment for the confounding variables. Furthermore, our study revealed that the RC/HDL-C ratio is the best predictor of GDM risk compared with conventional lipid profile TG, TC, LDL-C, HDL-C, and non-conventional lipid index RC. Meanwhile, through sensitivity analyses, we noticed that the RC/HDL-C ratio was associated with GDM risk among Korean women with a BMI < 25 kg/m^2^ or without hepatic steatosis. Several variables were considered to identify potential confounders that can affect the relationship between RC/HDL-C ratio and GDM events, including age, pre-pregnancy BMI, parity, hepatic steatosis, and HOMA-IR. The results revealed that RC/HDL-C ratio and GDM risk were unaffected by the aforementioned confounding variables, suggesting the robustness of our results.

Conclusively, the mechanism that links the RC/HDL-C ratio to GDM development is unclear and can take several forms. (1) Common pathway of metabolic diseases: RC carries a large amount of cholesterol toxic to PBC, inducing apoptosis and affecting insulin formation and secretion; HDL has cholesterol efflux capacity; RC and HDL-C participate in IR via opposing inflammatory effects. (2) Previous studies reported that the TG/HDL-C ratio is a valid surrogate marker for IR, with good IR prediction performance [76]. Additionally, most laboratories utilize enzymatic methods to detect TG levels, which not only measure the TG in the aforementioned lipoproteins but also free glycerol. Therefore, we cannot directly replace RC with TG. Abnormalities in TRLs (mostly RC-carrying lipoproteins) carried by apolipoprotein B in T2DM may precede IR [77]. Moreover, it has been indicated that RC particle diameter is associated with hemoglobin A1C. Similarly, RC was negatively correlated with HDL-C levels [78], suggesting that low HDL-C can be a biomarker for increased TG and RC levels. Therefore, selecting the RC/HDL-C ratio may be more favorable. (3) Sex-specific: Pregnancy increases circulating pregnancy hormones (including human placental lactogen, progesterone, and estrogen), which change normal homeostatic glucose pathways in the brain and pancreas, leading to impaired insulin sensitivity in maternal peripheral tissues [54]. Estrogen secretion during pregnancy promotes atherosclerotic lipid abnormalities, visceral weight gain, and IR, which increase the risk of liver disease and cardiometabolic disease [56, 79]. Moreover, the human placenta plays a crucial role in transforming cholesterol into steroids. The production of these hormones is vital for sustaining pregnancy and supporting the embryo’s growth. During the second half of pregnancy, IR status can be triggered by placental hormones [80], which potentially explains the role of RC in GDM development. (4) HDL participates in the reverse remnant-cholesterol transport. An earlier version of this hypothesis explained the adverse relationship between plasma HDL-C and CVD (free cholesterol transfer is impaired at low and very high triglyceride lipoproteins) [81]. Generally, a steady-state concentration of HDL-C can serve as a biomarker for cholesterol removal from TRLs in plasma. Some of the RC were lipolysis products (apolipoprotein A-I (apoA-I) and cholesterol), which generated HDL-C in plasma (50% of their species). This pathway originates from the intestine, with apoA-I produced via RC lipolysis and cholesterol and subsequently transported to the plasma via lymph. Accordingly, the plasma concentration of HDL-C represents an imperfect static measure of cholesterol flux through this dynamic pathway. Therefore, high plasma HDL concentrations may be both a result and a cause of the effective clearance of plasma RC. It is suggested that the RC/HDL-C ratio can reflect the dynamic process of this pathway, indicating a mechanism by which HDL facilitates the clearance of RC particles from the bloodstream, thereby contributing to overall lipid homeostasis. This clearance is crucial because RC particles, if accumulated, can have atherogenic and pro-inflammatory effects [82]. All these need to be demonstrated through our further research.

The present study has some advantages. Firstly, the relationship between the RC/HDL-C ratio and GDM has been demonstrated for the first time, not only in terms of the IR mechanism to explain it, but also in the direction of the reverse remnant-cholesterol transport doctrine to try to explain the dynamics of the partial transformation between RC and HDL-C. Secondly, it explains to some extent the similar but independent mechanisms of GDM and T2DM, which of course has been claimed at the genetic level. Furthermore, Mendelian randomization studies have revealed that apolipoprotein B is more dynamically representative of the association of TRLs and LDL-C and coronary heart disease (CHD). Besides, RC and HDL-C contain a wide range of apolipoproteins with different compositions and contents, suggesting that apolipoproteins can be the direction of our future research.

This study has certain limitations. First, the sample size was small, and the sampling population also had limitations, necessitating its validation in different populations. Second, this is a secondary analysis of a prospective cohort study based in Korea, which has inherent limitations, including a lack of control over the design and data collection of the original study in the context of secondary data analysis. Insufficient life and clinical information, including participant’s dietary intake and physical activity levels, can be confounding factors affecting the experiment’s outcome. Additionally, standardization of specimen collection and quality control during laboratory testing reflects the challenges of this study. Furthermore, excluding participants with a BMI ≥ 25 and hepatic steatosis strengthened the robustness of our study results but also presented potential limitations. Primarily, excluding relevant participants can limit the generalizability of our findings to broader populations where overweight, obesity, and hepatic steatosis are common. Excluding such participants could overlook the complex interplay of these metabolic factors and their impact on GDM risk. Moreover, given the rising incidence of obesity and related metabolic conditions globally, our criteria can underrepresent a significant demographic at risk of GDM. Moreover, RC measurement adopts a calculation method. Although the calculation method strongly correlates with the homogeneous reagent method, the detection result can be high because some intermediate products are counted, and the detection results are unreliable when the TG content is high [83]. Accordingly, the direct methods of measuring RC can give different results, which could affect the study results. Meanwhile, in this experiment, the two-step OGTT method was employed to diagnose GDM in the Korean population, which differs from the diagnostic criteria of the one-step OGTT method recommended by the IADPSG in other parts of Asia (including China). The database referenced in this article used the ACOG diagnostic criteria for GDM 2013, which solely confirmed the presence of GDM without specifying individual blood glucose values for the two-step testing method. Therefore, redefining GDM in this study using the updated 2018 ACOG diagnostic criteria poses a challenge. However, the minor discrepancies between the 2013 and 2018 criteria likely exert minimal influence on the study’s outcomes. Consequently, the results can differ, which requires further categorization and exploration in the future. Additionally, insulin secretion is limited in Asians compared with Caucasians, and PBC dysfunction is a significant risk factor for GDM in Korean females [64]. Furthermore, there is no dynamic monitoring of the entire gestational cycle to learn about the dynamic changes in this indicator during pregnancy. Finally, some studies have found that compared with patients with GDM having normal glucose tolerance, women with insulin sensitivity defects had higher fasting blood glucose and greater birth weight (thereby a higher probability of adverse events), whereas women with insulin secretion defects were less different from normal pregnant women [84]. Considering the heterogeneity in content and components between fasting and postprandial in RC, the role of the emerging lipid marker RC/HDL-C ratio in GDM and its metabolic subtypes deserves further exploration.

## Conclusion

This research emphasizes the initial demonstration of RC/HDL-C ratio’s efficacy during early pregnancy for predicting the development of GDM during the second trimester. Consequently, the RC/HDL-C ratio can be used as a useful tool for early screening and included in regular obstetric clinical evaluations. Prevention of at-risk pregnancies based on the RC/HDL-C ratio early in pregnancy has the potential to reduce the incidence of GDM and improve pregnancy outcomes. This study has the potential to reduce the burden of care at a medical level and the economic burden at a social level for patients with GDM, and can be effective in improving adverse maternal and neonatal outcomes in the long term.
